# Analytical approaches for myocardial fibrillation signals

**DOI:** 10.1016/j.compbiomed.2018.07.008

**Published:** 2018-11-01

**Authors:** Balvinder S. Handa, Caroline H. Roney, Charles Houston, Norman A. Qureshi, Xinyang Li, David S. Pitcher, Rasheda A. Chowdhury, Phang Boon Lim, Emmanuel Dupont, Steven A. Niederer, Chris D. Cantwell, Nicholas S. Peters, Fu Siong Ng

**Affiliations:** a ElectroCardioMaths, Imperial Centre for Cardiac Engineering, National Heart & Lung Institute, Imperial College London, United Kingdom; b Department of Aeronautics, Imperial College London, United Kingdom; c Division of Imaging Sciences and Bioengineering, King's College London, United Kingdom

**Keywords:** Atrial fibrillation, Ventricular fibrillation, Analysis, Voltage mapping, Rotors, Phase analysis, Shannon entropy, Dominant frequency, Organizational index, Complex fractionated electrograms

## Abstract

Atrial and ventricular fibrillation are complex arrhythmias, and their underlying mechanisms remain widely debated and incompletely understood. This is partly because the electrical signals recorded during myocardial fibrillation are themselves complex and difficult to interpret with simple analytical tools. There are currently a number of analytical approaches to handle fibrillation data. Some of these techniques focus on mapping putative drivers of myocardial fibrillation, such as dominant frequency, organizational index, Shannon entropy and phase mapping. Other techniques focus on mapping the underlying myocardial substrate sustaining fibrillation, such as voltage mapping and complex fractionated electrogram mapping. In this review, we discuss these techniques, their application and their limitations, with reference to our experimental and clinical data. We also describe novel tools including a new algorithm to map microreentrant circuits sustaining fibrillation.

## Highlights

• Mechanism of atrial and ventricular fibrillation remains poorly understood.
• Rotational drivers of fibrillation can be mapped using phase analysis.
• Voltage mapping can identify fibrotic substrate of fibrillation.
• Conduction delay algorithm identifies lines of conduction block at reentry core.
• Substrate and driver mapping is limited by resolution of clinical data sets.

## 1 Introduction

Atrial fibrillation (AF) and ventricular fibrillation (VF) are complex arrhythmias, and the electrophysiological mechanisms that underlie these arrhythmias are still incompletely understood. For both AF and VF, there is debate as to whether these arrhythmias are entirely disorganized rhythms, sustained by multiple wavefronts, or if they are sustained by an organized driver with fibrillatory conduction distant from the driver [1]. The ongoing uncertainty about the mechanisms of myocardial fibrillation is in part due to the limitations of currently available tools used to record fibrillatory data, such as limits in resolution, but is also largely due to the challenges in processing and interpreting the complex signals seen in myocardial fibrillation.

The commonly used methods to record signals during myocardial fibrillation in the experimental electrophysiology laboratory include recording electrograms using contact electrodes [2], or recording optical signals non-invasively in preparations loaded with potentiometric dyes [3]. In the clinical electrophysiology laboratory, contact electrogram recordings are most commonly used [4], although non-contact electrograms can also be recorded, aided by inverse solution mathematics [5].

Because of the complex nature of myocardial fibrillation, these recorded electrical or optical signals often appear chaotic, and the periodicity and organisation within the signal can be difficult to appreciate from the raw data. The challenge of any analytical approach used to handle fibrillatory data is to accurately translate what appear to be chaotic and disorganized signals into interpretable information about the nature of the propagating wavefronts and the underlying myocardial substrate sustaining the fibrillation.

In this review, we will first briefly summarize our current understanding of the mechanisms of AF and VF. We will then describe in detail, the utility and application of a number of analytical techniques used to process myocardial fibrillation data, including commonly used techniques such as dominant frequency (DF) analysis, Shannon entropy (SE), phase mapping, voltage and complex fractionated atrial electrogram mapping, illustrating these techniques with our own experimental and clinical data. In addition, we will also describe novel analytical tools developed in our laboratory, including methods for identifying lines of conduction block in cases where fibrillation is sustained by microreentrant circuits.

## 2 Current understanding of mechanisms of myocardial fibrillation

### 2.1 Atrial fibrillation

Our understanding of AF mechanisms has evolved over time. In the early 1900s, a “single focus” theory was proposed, whereby a rapidly firing single focus was postulated to produce atrial flutter or AF depending on the rate [6]. Thereafter, Moe et al. in 1960s, using a computer model of impulse propagation in a non-uniform two dimensional structure, described AF as being sustained by random self-perpetuating multiple wavelets throughout atrial tissue [7]. A study by Allessie et al. provided first experimental evidence of the multiple wavelet hypothesis showing that the coexistence of 4–6 wavelets was necessary for maintaining AF perpetuation in a canine heart model [8]. However, no rigorous proof exists to support the theory that a critical number of wavelets are required to maintain fibrillation. The contrary hierarchical hypothesis was supported by work by Jalife et al. in the 1990s that stated AF was sustained by highly organized drivers in the form of spiral waves or rotors that self-perpetuated through wavefront and wavetail interactions at a phase singularity point, without a need for an anatomical obstacle [9]. A phase singularity point resides at the core of a rotor and represents an area without a definite phase surrounded by neighbouring areas that show continuous cycling between –π to +π in a time and space dependent signal [1]. More recently, there has been increasing interest in the endo-epicardial dissociation hypothesis, which proposes that complex fibrillation is sustained by electrical dissociation of the different layers of myocardial tissue in the atrium, allowing waves to break through from one layer to another, increasing the complexity of fibrillation and the effective area through which it propagates [10].

### 2.2 Ventricular fibrillation

There is a paucity of data on VF mechanisms in human due to its short-lived and lethal nature. Our understanding of mechanisms involved in its genesis and perpetuation remain incomplete and is largely based on animal experiments or extrapolation from mechanisms described in AF. Moe's original multiple wavelet theory of AF was also found be applicable in VF in some earlier studies [11]. VF has frequently been described as a form of spatio-temporal chaos with ‘quasiperiodic transition to chaos’ whereby an initial primary spiral wave or re-entrant driver degenerates to a period of alternans and finally complete chaos [12]. In patients undergoing cardiac surgery this theory has been demonstrated to some extent. VF induced on cardiopulmonary bypass has been shown to demonstrate a degree of spatiotemporal organization at onset that degenerates and becomes more disorganized as it evolves [13]. The contrary hypothesis that VF is an organized phenomenon is also supported by some data in the literature. Organized drivers in non-ischaemic myocardial tissue and disorganized activation in ischaemic and border zone area have been described in animal models [2,14]. Spiral wave rotational drivers have also been shown in *in silico* models and hypothesised to be the primary driver [15]. Intramural re-entry has been described in *ex vivo* perfused human hearts at sites of fibrosis [16], whilst mapping with basket catheters has demonstrated spiral wave re-entry during VF induction in ventricular tachycardia ablation procedures [17].

## 3 Approaches to mapping sources of myocardial fibrillation

### 3.1 Dominant frequency

Fibrillation data, whether recorded directly as voltage readings by contact electrograms or indirectly by fluorescence on optical mapping is a time series of a changing signal. The hypothesis is that, in complex fibrillatory signals, which lack clearly identifiable cycle lengths due to constant amplitude and frequency changes, the areas with the highest dominant frequencies represent areas that are driving the fibrillation, and by abolishing it, the process can be terminated [18].

The dominant frequency of a signal is defined as the frequency with highest energy in the power spectrum. Practically, the discretely sampled time-domain signal is transformed into the frequency domain using a discrete Fourier transform (or fast Fourier transform) and the relative power of each frequency is calculated. For a spectrum *X*(*f*), the dominant frequency is defined as the frequency of highest amplitude, i.e.,

$$DF=arg\;\underset{f}{max}X\left(f\right)$$

#### 3.1.1 Dominant frequency and rotational drivers

**Fig. 1:**
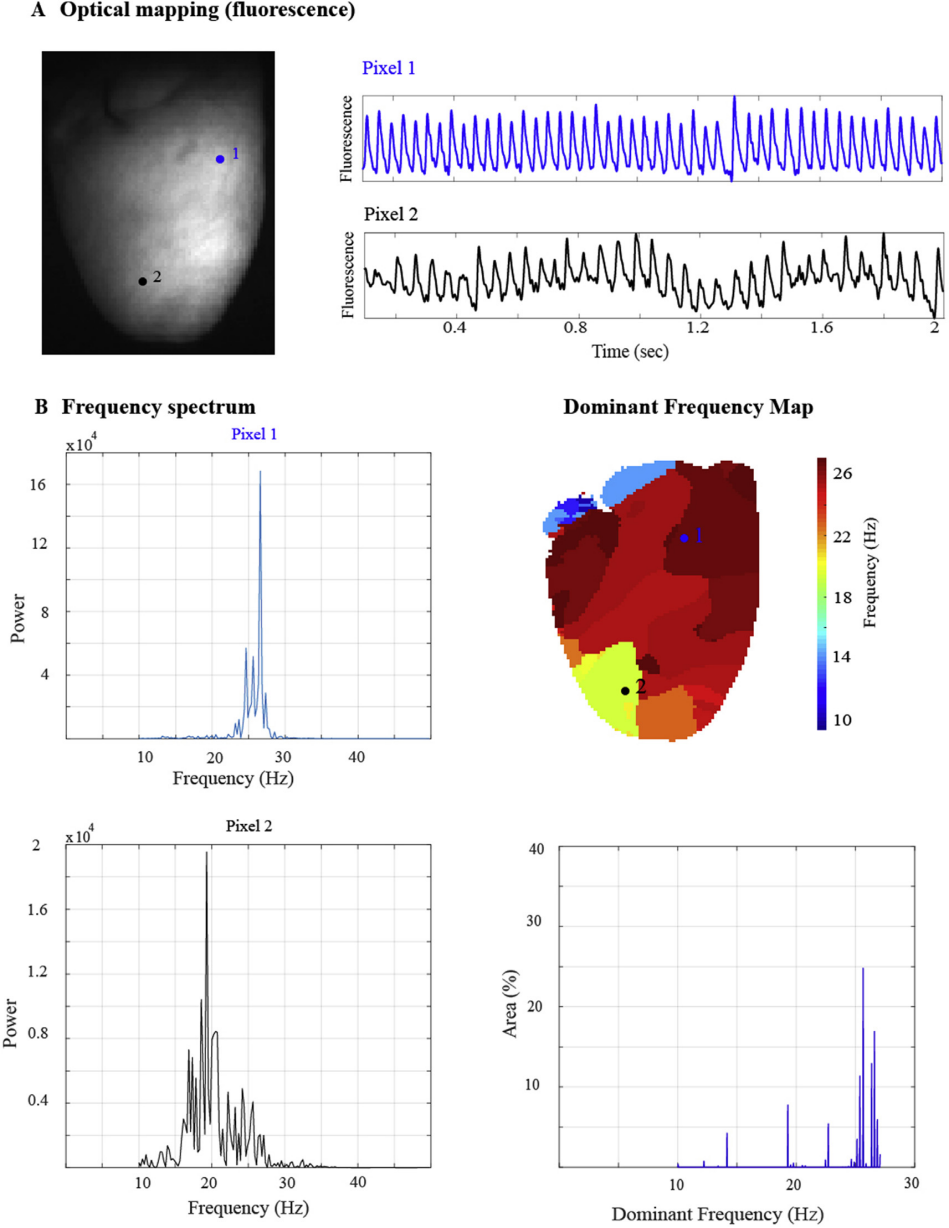
**Dominant Frequency Maps of VF: (A)** Optical action potential data for two pixels during a recording of ventricular fibrillation in a langendorff perfused rat heart. **(B)** Dominant frequency map: frequency spectrum of both these pixels is shown (left) and the frequency with the largest power for each pixel is plotted on a colour map. The corresponding histogram of DF values for the entire heart is also shown (bottom right). (For interpretation of the references to colour in this figure legend, the reader is referred to the Web version of this article.)

Sites of high DF compared to their surroundings have been postulated to be sites of primary activation and localise to areas identified as sustaining rotational activity in fibrillation [19,20]. Simulation studies of stable long duration rotors termed ‘mother rotors’ have demonstrated highest DF values at the core [21]. Fig. 1A and B demonstrate an example of a DF map in rat VF. The frequency spectrum of the optical signal at each pixel was calculated. The frequency with the greatest power was designated as the DF of that pixel and a DF map was created. In this example, there was a spectrum of frequencies between 10 and 27 Hz with a DF of 26 Hz. The highest or maximal DF in this example occupied a large spatial area rather than a discrete point that identified a primary activation site.

#### 3.1.2 Dominant frequency in clinical experience

Electrograms have been used to construct DF maps of the atrium, with areas of maximal DF subsequently targeted for ablation. In a randomised control trial designed to assess the effectiveness of targeting high- (dominant) frequency source ablation (HFSA) in AF, this approach was not superior to conventional treatment [22]. However, global DF measures, such as an overall reduction in the maximal DF, have been associated with reduced AF recurrence and abolishing baseline LA to right atrium (RA) DF gradients that often exist in AF with ablation has been associated with long term maintenance of sinus rhythm [23]. In our rat VF experiments, whilst we observed higher relative DF values in areas of rotational drivers, areas with high DF were also seen at areas without drivers, suggesting limited specificity of DF mapping for fibrillatory drivers.

### 3.2 Organizational index

DF maps in AF have been found to be spatiotemporally unstable and ‘high’ DF values spuriously result from wave front collision in areas remote from rotational activity, calling into question feasibility of DF guided ablation. Our group has previously postulated that sites with high DF organization, termed the organizational index (OI), defined as mean ratio of the power of the DF and its harmonics (within a 0.75 Hz window) to the total power of the spectrum, may be sites of AF drivers.

In persistent AF (PsAF) patients, ablation of sites of high OI resulted in an increase in the OI of areas remote from these sites (left atrial appendage), supporting the focal source hypothesis [24]. Areas of high PS clustering were found to correlate with areas of high OI and there was an increase in OI after circumferential pulmonary vein isolation (PVI) [25]. Although interest in DF guided ablation has waned, there might be a role in utilising it with a more spatiotemporally stable methodology such as OI to identify substrate.

### 3.3 Shannon entropy

Entropy is a measure of unpredictability of information content. Shannon entropy (SE) is a measure that calculates the smallest number of bits to communicate an amount of information losslessly. Signals that are predictable, for instance, sinus rhythm on an ECG, have low arbitrary entropy values whereas those that are more variable such as VF on ECG would have high entropy values [26]. SE can be applied to electrogram or optical mapping data from different spatial locations within a ventricle or atrium to construct SE maps and by discerning areas of high and low entropy, driver regions may be identified. SE is calculated by binning the signal using a predefined bin size that is amplitude dependent. The value of SE for signal is then calculated using the relative probability (Pi) of the signal falling in each amplitude bin (N). The equation applied to an electrogram is:

$$SE=\sum_{i=1}^{N-1}Pi\cdot log_{2}Pi$$

#### 3.3.1 Shannon entropy and rotational drivers

**Fig. 2:**
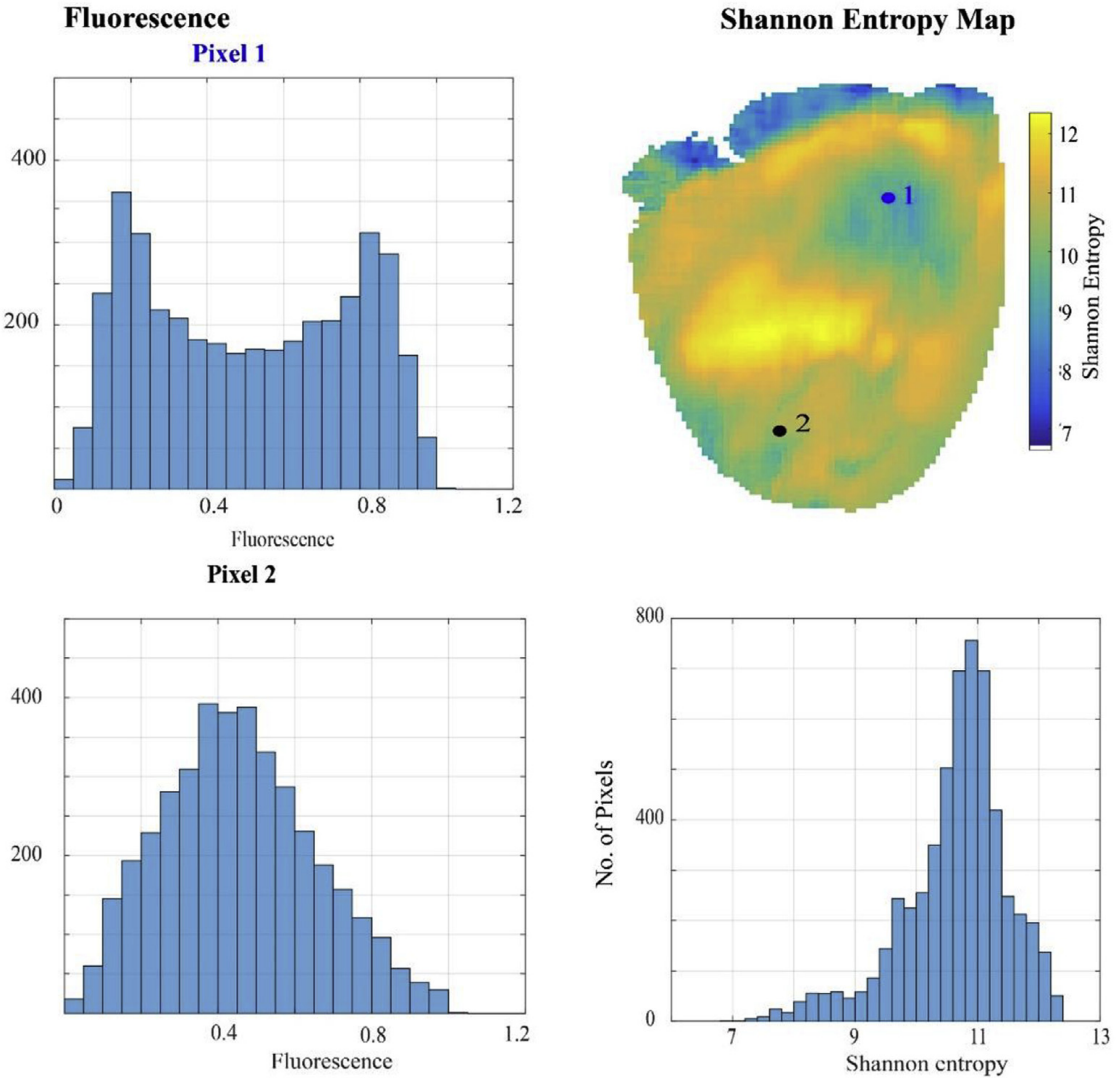
**Shannon Entropy Maps of VF:** The optical action potential data for the two pixels during a recording of ventricular fibrillation shown in Fig. 1 was used to construct a Shannon entropy map. The fluorescence signal from optical mapping of each pixel is placed in fluorescence amplitude bins with frequency of occurrence over the recording period on the y axis (left), and the Shannon entropy of each pixel was calculated using the relative probability of the signal falling in each fluorescence amplitude bin. The Shannon entropy values for each pixel are then depicted on a Shannon entropy map (top right), the distribution of SE values shown on the histogram (bottom right).

The primary objective of applying SE to fibrillation data is to identify rotational activity or drivers either from its value in a single area relative to others, or from calculation of a SE gradient [26]. In animal AF and VF models, SE has been applied to optical mapping signals to show a gradient in values with higher SE values occurring at the core of the rotational activity relative to the surrounding regions. The pivot zone at the core has a more complex fragmented signal with high SE values, whereas further from the core, activation is more regular and periodic with lower SE values. Ganesan et al. showed this inverse correlation between SE and increasing distance from the pivot point/rotational core using paired optical mapping and bipolar electrogram data in isolated rat and sheep atrial preparations [27]. Other animal studies have shown a similar gradient but with a contradicting relationship with a low SE at the core of rotational activity [26,28]. Fig. 2 demonstrates an example of a SE map for our rat model of VF using the method described above. In our experience, when applied to optical mapping data, SE is not able to accurately localise rotational driver regions identified with phase analysis.

#### 3.3.2 Shannon entropy in clinical experience

There is a paucity of SE processed human AF data and its use remains in its infancy. Limited mapping studies have shown areas of high SE outside pulmonary veins in PsAF [28]. Only recently, has the feasibility of using SE and other forms of entropy analysis (Renyi and Multiscale Entropy) with 3D LA mapping of AF been explored to identify the potential substrate for ablation [26].

### 3.4 Phase mapping

Phase mapping is a technique for localizing phase singularities that exist at the core of a rotational driver [29]. Over the last two decades, there has been a lot of interest in the hypothesis that ablating tissue in areas that localise phase singularities might be the critical step in treating fibrillation. Some initial clinical studies showed favorable results for long term freedom from AF with rotor-guided ablation [30], though many subsequent studies have shown disappointing results [31,32,33].

#### 3.4.1 Identifying spatiotemporal organization with phase analysis

Despite fibrillation being a seemingly random process, Gray et al. developed a technique to identify a degree of temporal organization and periodicity in fibrillatory signals [34]. They used a technique from nonlinear dynamics called state-space mapping, in which the original signal (the first state variable) is plotted against a time-delayed version of the signal (the second state variable). If there is a consistent dependence of the future samples of the signal on past samples, this will manifest as loops around a central attractor in the coordinate system defined by the two state variables [35]. The phase angle then describes the angle as the system moves through this loop or trajectory. This phase angle is measured at each spatial and temporal location to give a spatial phase map, and a spatial singularity in phase occurs at the center of rotating waves. This landmark paper revealed a degree of spatiotemporal organization in fibrillation and the technique used to reveal this organization is one method that can be used to locate the tip of spiral waves and analyze their dynamics.

#### 3.4.2 Current techniques for phase analysis

Current measurement techniques record a single variable, typically either the extracellular potential or membrane action potential. The phase angle requires the choice of an appropriate second variable. Early studies utilising phase mapping plotted the optical mapping fluorescence signal (used as a surrogate for the membrane potential) against a time shifted fluorescence signal [34]. Whereas a particular membrane potential could refer to either depolarization or repolarization, the phase angle gives a unique value for each point in the action potential [36]. Phase has the additional advantage that the great variability, present during arrhythmia, in action potential duration and cycle length, does not affect the state-space map [37]. Each action potential results in the phase angle preceding through a 2π trajectory, and subthreshold stimuli do not result in loops in the state-space, so long as the time shift and origin of the map are chosen correctly.

**Fig. 3:**
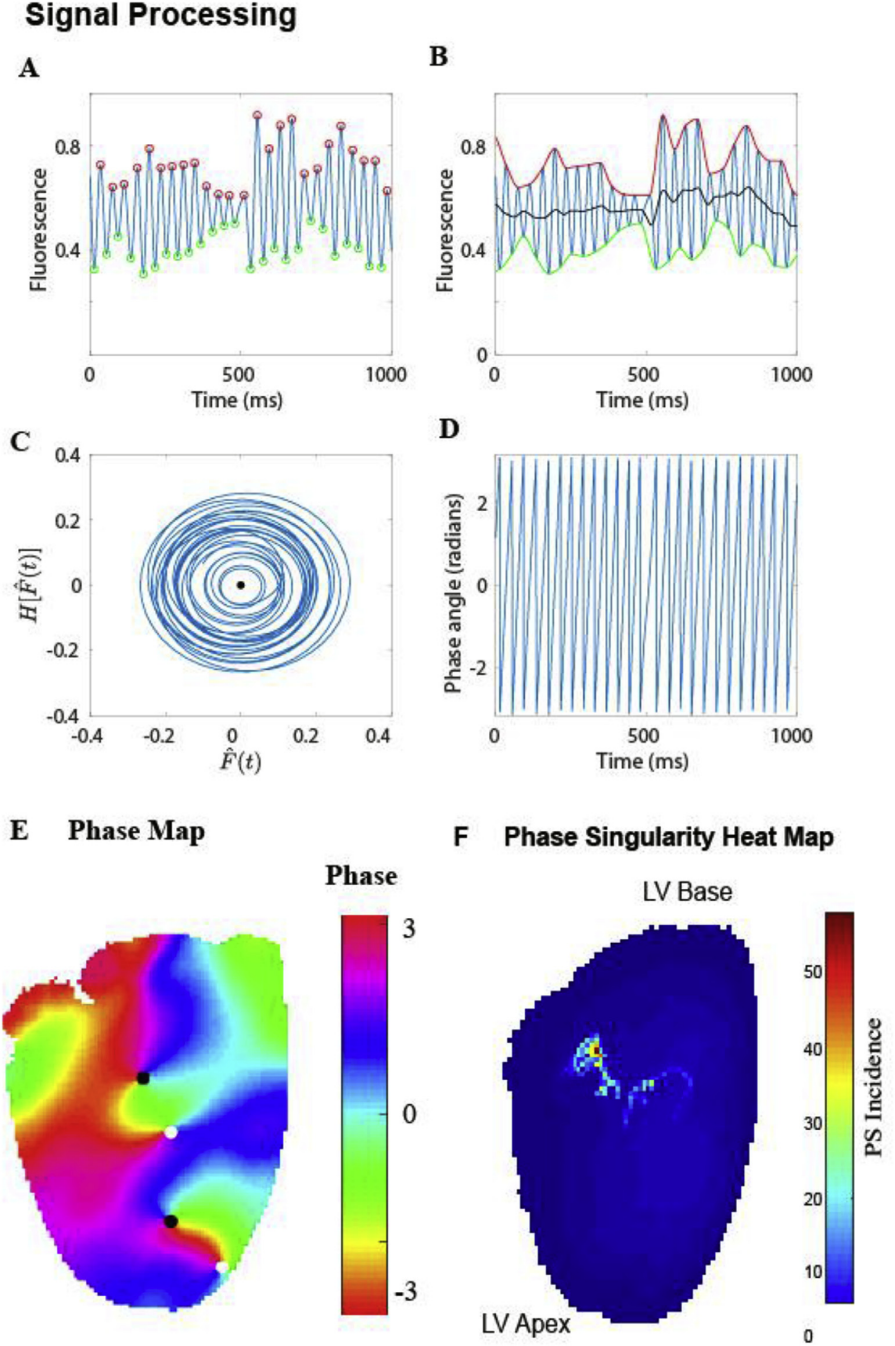
**Calculating phase in optically mapped VF: (A**–**F)** Steps for calculating the phase are as follow. **(A)** Tag minima (green circles) of the signal using a sliding window, then tag maxima (red circles) between each pair of minima, and finally remove any small amplitude pairs of maxima-minima. **(B)** Cubic spline fits were performed on the maxima and minima of the signal, and the average of these maxima (red) and minima (green) splines (the mean line, shown in black) was subtracted from the signal to give a signal of zero mean. **(C)** The real and imaginary parts of the Hilbert transform of this zero-mean signal were plotted in the phase plane. **(D)** The angle around the trajectory in (C) gives the phase angle. A phase map of ventricular fibrillation at a single time point is shown in **(E)**, and a phase singularity heat map can be used to show sites with high incidences phase singularities over time **(F)**. (For interpretation of the references to colour in this figure legend, the reader is referred to the Web version of this article.)

There is no consensus on the optimal way to choose an appropriate time shift to generate the second variable: too large a time shift results in overlapping state-space trajectories, while too small a shift means the signals are too similar [34,38]. Another choice of second state variable is the derivative of the first signal, which does not require the choice of a time delay, but is sensitive to noise [39]. More recent studies use the Hilbert transform, which is widely used in signal processing, to generate a suitable phase-shifted second variable for phase analysis [40]. Fig. 3 demonstrates an example of phase analysis in a rat model of VF using Hilbert transform, where over a 4 s recording, we were able to generate a heat map of tracked phase singularities, which localized to a discrete area near the base of the left ventricle.

A second challenge associated with phase mapping is that phase loops in the two variable phase space should progress around a central point from which the phase angle is calculated. Various techniques have been employed to generate a zero mean signal so that phase loops encircle the origin of the two variable phase space. For example, Bray and Wikswo developed a pseudo-empirical mode decomposition technique in which a line calculated as the mean of maxima and minima splines is removed from the signal to produce a zero mean signal [37]. They used a moving window for assigning maxima and minima of a length equal to half of the average cycle length, with the motivation of detecting double potentials that may occur during reentry. Linear and quadratic detrending techniques have also been employed to generate zero mean signals [41,42].

Spatial maps of phase may be processed to identify phase singularities at which there is a 2π progression in phase in the surrounding points and the phase value is undefined [43]. Phase singularities may indicate rotational activity, wavefront break-up or conduction block [44]. Analyzing the locations of phase singularities over time is one technique that can be used to reveal the path of rotational activity. Topological rules enforce that the ends of wavefronts must either be connected to each other to form loops, to boundaries or to phase singularities [42].

#### 3.4.3 Application of phase mapping

Phase mapping was originally applied to transmembrane potential data. More recently, techniques have been developed for phase mapping of unipolar and bipolar electrogram data, during both VF [41] and AF [40,45,46,47]. The correct calculation of electrogram phase during AF is difficult due to several factors, including the non-sinusoidal and fractionated nature of the electrograms, and the direction-dependency of bipolar electrograms. Kuklik et al. developed a phase mapping technique using sinusoidal recomposition for atrial unipolar electrograms in which the signal is represented as a sum of sinusoidal wavelets with amplitude proportional to the negative slope of the electrogram [40]. This pre-processing generates a sinusoidal signal suitable for phase mapping using the Hilbert Transform. We previously applied a sequence of filters typically used for DF analysis to either bipolar electrograms or the derivative of unipolar electrograms to make the signal more sinusoidal, together with a pseudo-empirical mode decomposition technique to create a zero mean signal, before application of the Hilbert Transform [46]. Phase mapping may also be applied to noninvasively computed unipolar electrograms. The filter settings used prior to phase mapping will affect rotor detection [48,49].

Phase mapping does not assign particular importance to an activation point, which is advantageous for fractionated signals in which it is difficult to assign an activation time. Kuklik et al. compared cycle lengths calculated from times assigned to the unipolar signals to those calculated from the times of phase inversions and showed a good correlation [40].

**Fig. 4:**
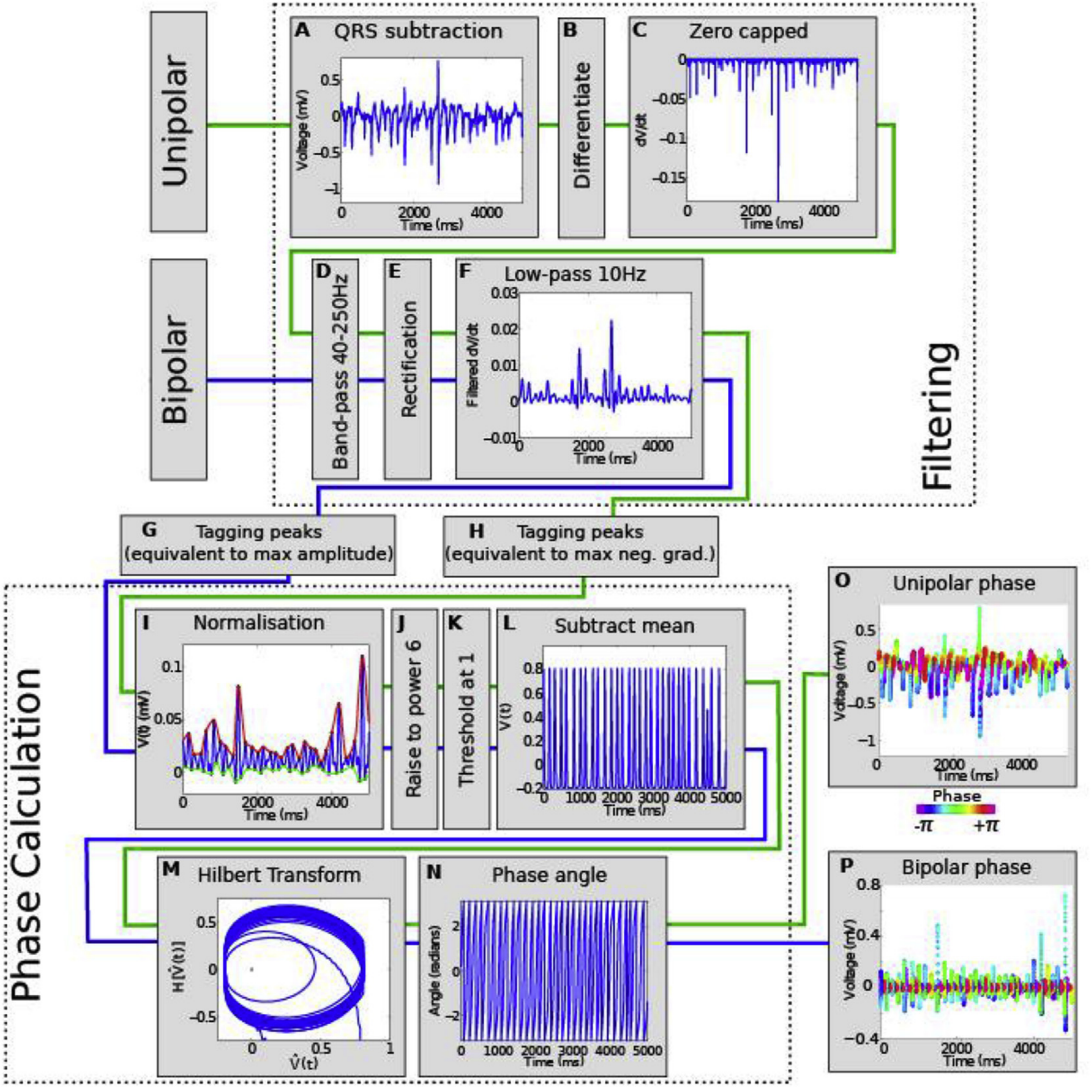
**Outline of filtering and processing steps involved in phase calculation of unipolar and bipolar electrogram signals.** Raw unipolar electrograms are pre-processed **(a**–**c)**. Both unipolar electrogram derivatives and bipolar electrograms are then pre-processed **(d**–**f)** before tagging of individual activations **(g**–**h)**. Phase is then calculated identically for both modalities of signal **(i**–**n)** to give unipolar and bipolar phase **(o**–**p).** [Figure reproduced from Roney et al. [46], in line with Creative Commons Attribution license (CC BY 4.0)].

**Fig. 5:**
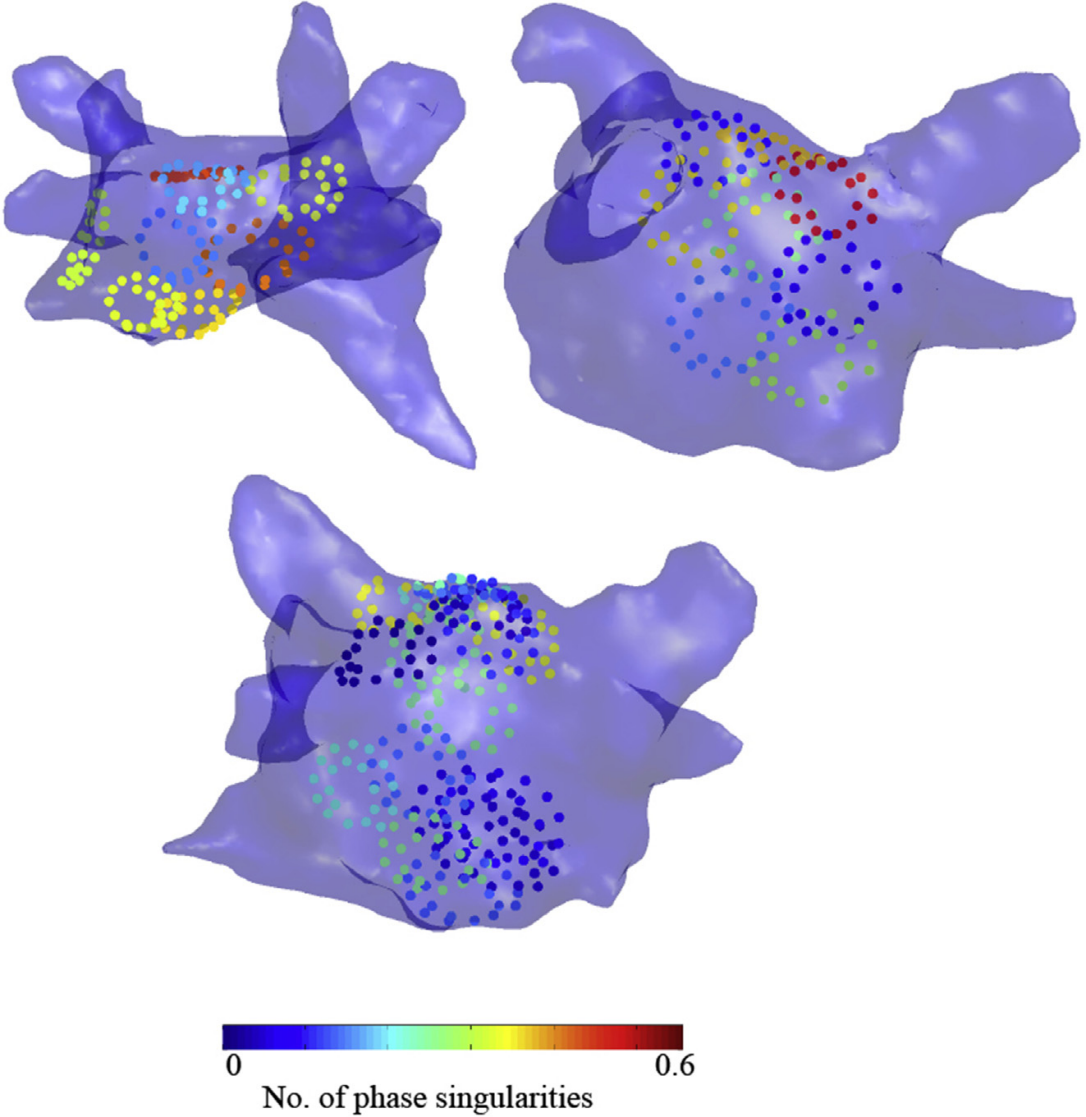
**Left atrial 3D reconstruction showing phase singularity heat maps during AF.** Regions of high and low phase singularity during AF are shown for three patients, with maps created using bipolar electrogram phase analysis. [Figure reproduced from Roney et al. [46], in line with Creative Commons Attribution license (CC BY 4.0)].

Fig. 4 shows our methodology for filtering and processing raw unipolar electrograms to calculate phase to track phase singularities. Using data from clinical multipolar catheters and electroanatomical mapping we are able to generate 3D reconstruction of the left atrium with regions of phase singularities super imposed (Fig. 5). The number of mapped phase singularities vary between different area of the left atrium and between patients. In our experience, both unipolar and bipolar electrograms are similarly effective in their ability to track phase singularities from clinical data sets [46].

#### 3.4.4 Impact of spatial resolution on phase analysis

Recording modalities are typically limited in either resolution or coverage. We recently investigated how spatial resolution affects interpretation of AF recordings, expressing spatial resolution requirements as a linear function of the spatial wavelength, and found that high-density multipolar catheters provide sufficient resolution, but basket catheters are prone to false rotor detections [50]. Aronis et al. considered the effects of multiple co-existing rotors on resolution requirements in an *in silico* study and found that including more than one rotor increased errors 10-fold, suggesting higher resolution requirements for cases with multiple sources [51]. Jacquemet used a statistical model to investigate the effects of interelectrode spacing and rotor number and showed an increase in false positive and false negative phase singularity detections with increasing interelectrode distance [52].

Phase mapping is a technique that is effective with the high spatiotemporal resolution of laboratory-based technique such as optical mapping, however with the limitations imposed by resolution and coverage of current clinical catheters, it is unsurprising that at present rotor guided ablation as an adjunct to conventional PVI has not been proven to be beneficial in a recent systematic review of 11 separate studies [53].

## 4 Approaches for mapping the fibrillatory substrate

As opposed to directly mapping the location of putative drivers of myocardial fibrillation, as described above, techniques exist to map the myocardial substrate sustaining fibrillation, which provides additional useful information.

### 4.1 AF voltage mapping and fibrosis

Voltage amplitude from contact electrograms is often used as a surrogate marker for scar, largely from data extrapolated from mapping in the ventricles, and in identifying diseased myocardium as part of a substrate based approach in catheter ablation of ventricular tachycardia [54]. In the clinical cardiac electrophysiology laboratory, attempts have been made to identify and target the anatomical substrate of fibrosis using electrogram voltage mapping. There is evidence to support the hypothesis that the presence and extent of low voltage areas can predict the outcomes of catheter ablation of AF [55]. Low-voltage areas in the atrium may represent regions of slow conduction as a manifestation of underlying fibrosis, and/or wave collisions representing more functional electrophysiological change and these regions have been purported to be crucial in the maintenance of AF. Slow conduction in diseased myocardium may be necessary for sustaining rotor activity, and localized rotational activity observed with multipolar endocardial mapping was found to co-localise with regions with very low voltages in AF (<0.1 mV) [56].

#### 4.1.1 Electroanatomical voltage mapping

Voltage maps are constructed in the clinical catheter laboratory using three dimensional (3D) electroanatomical mapping (EAM) systems. Contemporary EAM systems construct 3D shells of the cardiac chamber of interest by plotting ‘electrical points’ as they are sampled from the endocardial wall by a mapping catheter with an inbuilt sensor which is tracked in space with either a weak magnetic or electrical field. EAM systems depict voltage maps on these 3D reconstruction models as colour coded regions of dense scar, border zone and healthy tissue using voltage amplitude cut offs of <0.5 mV, ≤0.5–1.5 mV ≤ and >1.5 mV respectively, for the ventricles [57]. The voltage amplitude is measured typically as the potential difference between the peak negative and peak positive deflection (peak to peak) of a bipolar or unipolar electrogram over a defined time window [58]. Voltage maps can be created in sinus rhythm, paced rhythm or in AF. One of the challenges of creating voltage maps in fibrillation is that, due to complex nature of the electrograms, it can be difficult to identify the peak to peak deflections accurately and also voltage amplitude can change continuously in fibrillation and vary significantly in sinus rhythm from AF in the same region [59]. These observation in fibrillation are hypothesized to be due to cancellation of colliding wavefronts, functional/anatomical blocks [60] and varying wavefront curvature interaction with bipolar electrodes [61].

#### 4.1.2 Ablation of low voltage regions

Current substrate modification strategies targeting potential sites AF drivers involve targeting these low voltage regions. The spatio-temporal variability of voltage signals in AF underlies the challenges of voltage mapping in AF, and some have adopted an approach of mapping in sinus rhythm or during pacing to more accurately study the underlying substrate. Low voltage areas are more represented in persistent AF than in paroxysmal AF, in studies utilising voltage in the paced-rhythm [62], and in AF [63].

The notion of scar homogenization, once again, extrapolated from catheter ablation of ventricular tachycardia, where multiple ablation lesions are created within myocardial scar to eradicate all potential channels that support the arrhythmia [64] has now been described as part of a substrate modification approach in AF. Rolf et al. described a novel individualized approach, where catheter ablation of AF based on low-voltage areas in the left atrium improved clinical outcomes [65]. Left atrial voltage maps were created in sinus rhythm after circumferential PVI, and low-voltage areas defined to regions with bipolar endocardial voltages of <0.5 mV [66]. An ablation strategy was adopted with the aim of preventing substrate-based initiation and perpetuation by elimination of all reduced potentials of smaller areas, or by applying strategic linear lesions through larger low-voltage zones.

One of the limitations of voltage guided ablation strategies is the paucity of data to support the use of a bipolar 0.5 mV cut off as a surrogate for scar with modern multi-electrode catheters used in EAM systems [67]. This voltage threshold was based on data acquired in older studies using mostly ablation catheters in ventricular mapping studies. Recording electrode size, interelectrode spacing, tissue contact, mapping density and bipole orientation are all important determinants of voltage recordings and vary significantly with multi-electrode catheters [[67], [68], [69]]. As such the threshold amplitude of 0.5 mV for scar may itself need to redefined, especially in the atrium.

#### 4.1.3 Correlation of AF voltage maps with scar on MRI

Late-gadolinium cardiac magnetic resonance imaging (LGE-CMRI) has now been used to detect atrial fibrosis. The burden of atrial fibrosis as determined by LGE-CMRI positively correlates with clinical indices of atrial structural re-modelling and outcomes of catheter ablation [70], and with the number of sites exhibiting high rotor activity using non-invasive mapping, and rotor trajectories showed a clustering around borders of fibrotic areas [71].

**Fig. 6:**
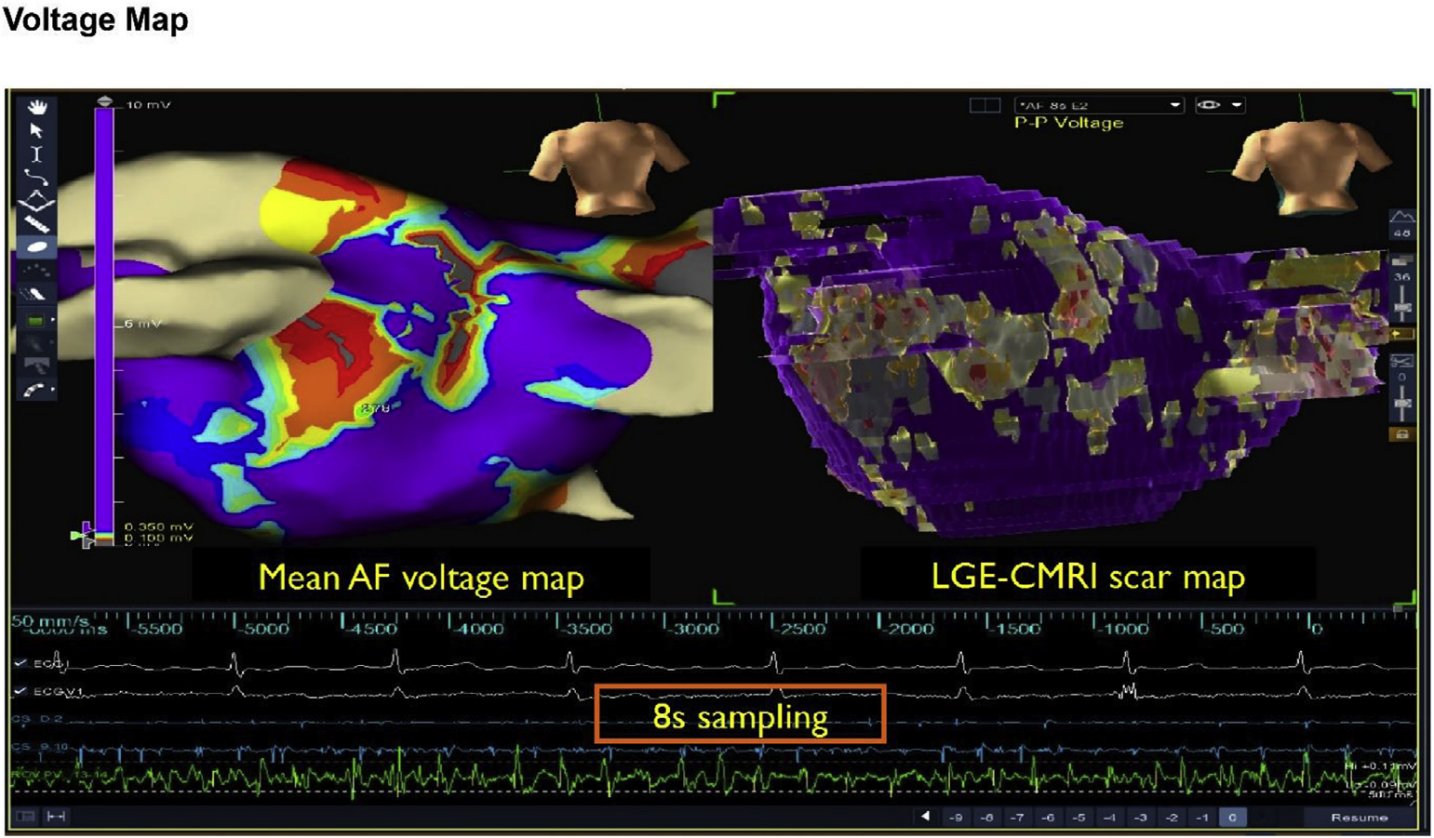
**AF voltage maps correlate well with MRI scar maps.** Electroanatomical voltage maps in AF (left), with their corresponding electrograms. The AF voltage maps were created using 8 s segments of recordings, and the maps represents the mean peak-to-peak AF voltage (AF-V) (on a research version of EnsiteTM Velocity, SJM). The LGE-CMRI derived atrial scar maps (right) are imported, and registered to the left atrial geometries. This representative example illustrates good correlation between regions of low mean AF voltage and scar as defined by LGE-CMRI.

We recently described a novel technique on assessment of the atrial substrate with the use of AF voltage [72]. Given that variable electrograms recorded during AF are generated by the dynamic interaction of local electrophysiology in response to the underlying myocardial architecture, we reported that, under conditions of adequate sampling duration of at least 4s, the spatial distribution of mean AF voltage is reproducible, and correlates with scar maps from LGE-CMRI (Fig. 6). Mean AF voltage holds promise as a unique marker reflecting the functional response to the underlying AF substrate.

### 4.2 Mapping complex fractionated atrial electrograms (CFAEs)

In addition to representing local activation, the content of a clinical electrogram in itself has been hypothesised to contain information in regards to the underlying fibrillatory substrate and mechanism. Konings et al. first described the complex fractionated atrial electrogram (CFAE) morphology in intraoperative studies in 1997 and from local activations maps demonstrated that they were representative of lines of conduction block, wavefront collisions, slowed conduction and pivot points for microrentrant circuits [73]. CFAEs are defined as low voltage atrial electrograms (ranging from 0.04 to 0.25 mV) that have fractionation consisting of two deflections or more, and/or consist of a prolonged activation complexes with perturbation of the baseline with continuous deflections. They may be associated with a short cycle length (<120 m s), form discrete complexes or fail to demonstrate isoelectric intervals between complexes [74].

#### 4.2.1 Mapping and ablating CFAEs

Nademanee et al. (2003) were first to create EAM of CFAE in AF and by applying radiofrequency ablation to areas with CFAEs managed to terminate AF in 95% of their patient group, consisting of both persistent and paroxysmal AF, and achieved a remarkable 91% arrhythmia free survival at one year follow up [75]. Subsequent trials have failed to show such favourable results [76,77].

The variable clinical outcomes have been attributed to a number of factors. CFAE mapping and ablation is performed in a very heterogeneous manner between different centres and there is a large degree of variance in inter-operator experience and targeting of CFAEs [78]. The commercially available automated EAM systems, namely NavX and CARTO, vary significantly in how their algorithms classify CFAEs and currently expert consensus on how CFAEs should be mapped is lacking [79]. Lastly, from a mechanistic point of view there is an argument that CFAEs are non-specific markers. Whilst they may take part in AF mechanisms, they can also be generated passively by mechanisms that do not contribute to AF [80]. Classification of CFAEs by mapping local refractoriness of atrial tissue has demonstrated that the majority of the CFAE may in fact be far field signals and the rest may represent rapid localized AF site, AF acceleration dependent CFAEs or localized disorganization [81]. At present, lack of specificity of CFAE is a concern and analytical tools for identifying true CFAEs contributing to AF mechanisms are lacking.

## 5 Novel tools for fibrillation analysis

In addition to mapping putative drivers and substrate, novel tools are being developed to provide unique mechanistic insight into fibrillatory mechanisms. There is increasing evidence that drivers may in fact represent microreentrant circuits [82], and we recently describe a method we developed to map the nature of such circuits during fibrillation [83].

### 5.1 Algorithm for identification of lines of conduction block in microrentrant circuits

One of the challenges of processing fibrillatory data, particularly at the microscopic scale, is to isolate features of interest so that they may be studied in relation to specific hypotheses. To analyze our recordings of rotational activity at single-cell level, we developed an algorithm to highlight areas of consistent conduction block or slowing over the course of the recording. This allowed us to generate ‘heat-maps’ of conduction block which can be studied in relation to features of the underlying substrate, such as cell size, ion channel density or gap-junctional coupling.

**Fig. 7:**
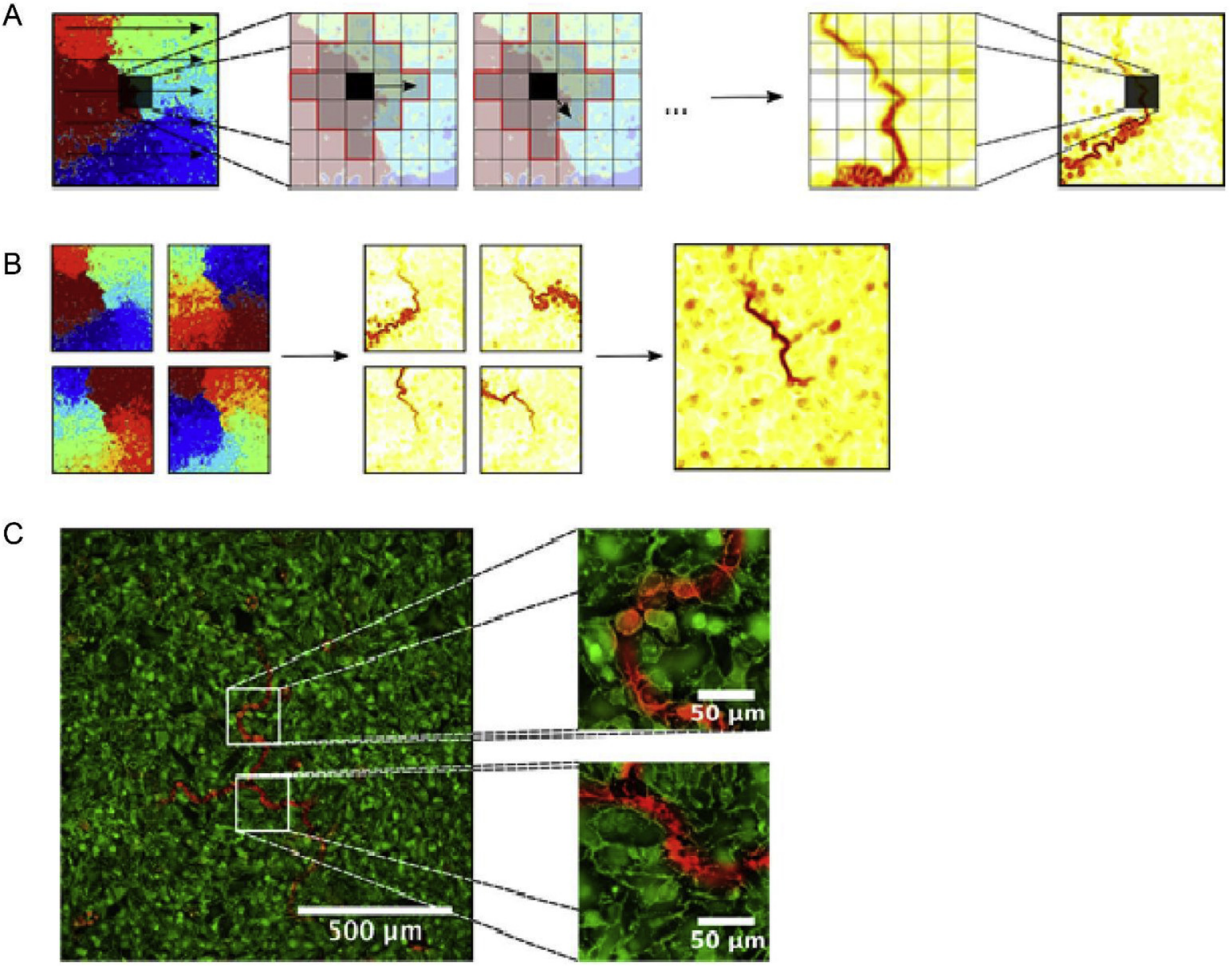
**Processing and analysis of conduction block at single-cell level in HL1-6 myocyte monolayer.** (**A)** Processing stage of algorithm: each pixel in the activation map is iterated through (first graphic). For each pixel, its neighbourhood is determined and then the absolute activation time difference between it and each neighbouring pixel determined (next two graphics). After repeating for all neighbourhood pixels for every pixel in the activation map, a heatmap highlighting areas of large activation differences is generated (last two graphics). **(B)** This process can be repeated for multiple activation maps generated from the same recording. When these heatmaps are summed and averaged, the resulting heatmap isolates the ‘constant’ features of the rotational activity. **(C)** The final heatmap can be overlaid on wheat germ agglutinin membrane staining to study the correlation between cell morphology, core shape and/or core location.

A pictorial outline of the algorithm is shown in Fig. 7. Firstly, each raw recording is processed using conventional techniques: spatial binning, temporal filtering and drift correction to reduce unwanted noise while retaining the underlying signal [36]. A series of activation maps are generated for the range of time points in the recording, each covering a single rotation of the circuit.

Areas of high activation time disparity are then mapped (Fig. 7A). For every pixel (i), neighbourhood pixels within a user-defined radius are found. For each neighbourhood pixel (j), the absolute activation time difference is calculated as $AT_{diff}=\left|AT_{i}-AT_{j}\right|$. This value is added to a running total at the geometric midpoint between i and j. After the values for every j have been calculated, the algorithm iterates onto the next i pixel in the activation map and the process repeats. After the whole activation map has been processed, the mean activation time difference at each pixel is then calculated to generate a heatmap which highlights pixels between regions of large activation time differences. This generally corresponds to the wavefront itself and the core (as shown in Fig. 7B).

The process is repeated for all activation maps in the recording, and the mean values through time calculated for each pixel. This has the effect of retaining only ‘constant’ features in the recordings (i.e. stable core) and eliminating features that change from frame to frame (i.e. the wavefront of activation). These maps have revealed that cores of rotational activations at microscopic level can be composed of *lines* of conduction block or slowing up to a size of ∼1.5 mm, rather than propagation occurring around a single point.

This map can be aligned and overlaid on further microscopy images to correlate the core of rotation with features to test specific hypotheses. As an example, we show in Fig. 7C the core heatmap overlaid on fluorescently labelled wheat germ agglutinin membrane staining to study the effect of differences in 2D cell size to the location and morphology of the core. Zoomed portions of the full image show the level of detail we are able to achieve with this approach.

Using this technique, we recently demonstrated existence of rotational activity at a cellular scale [83]. We studied features of the core of the rotational activation at single-cell level resolution (2.6μm/pixel) in a homogeneous substrate (2D monolayers of the HL1-6 cell line [25,84]) using fluorescence microscopy. We observed rotational activity that remains stable for periods of several minutes despite the homogeneity of the substrate, and rotates around a core that does not show the features of a phase singularity (when imaged with both Ca2+ and voltage sensitive dyes) [83]. Our conduction delay algorithm showed that the cores of rotational drivers in HL1-6 cell line consist of areas with lines of conduction block. These data suggest that the rotational activity is sustained by micro-reentrant circuits, and not by functional reentry or spiral waves. Other conventional methods such as phase mapping would merely assign a phase singularity to the core of the rotational driver and not be able to distinguish between micro-reentry, functional reentry and spiral waves at the cellular level.

## 6 Conclusion

Analysis of myocardial fibrillation data presents a major challenge. Analytical techniques exist to map putative drivers of fibrillation or to map the underlying myocardial substrate sustaining fibrillation, and data analysed using these techniques, especially in the experimental laboratory, have provided important insights into the mechanisms of AF and VF. Nonetheless, many of these techniques can be limited by the resolution of the mapping data, especially in the clinical setting, and the success rates of therapeutic ablative approaches reliant on these techniques remain modest. There remains an urgent need to develop novel analytical tools to process myocardial fibrillation data.

## Funding

The work is funded by the British Heart Foundation (grants number RG/16/3/32175, PG/15/59/31621 and PG/16/17/32069, and British Heart Foundation Centre of Research Excellence Studentships RE/13/4/30184), the Wellcome/EPSRC Centre for Medical Engineering (WT, 203148/Z/16/Z), the Imperial College Centre for Cardiac Engineering and the National Institute for Health Research Imperial Biomedical Research Centre.

## Authors

Balvinder S. Handa, BSc MRCP, Caroline H. Roney, PhD, Charles Houston, MRes, Norman A. Qureshi, MRCP PhD, Xinyang Li, PhD, David S. Pitcher, PhD, Rasheda A Chowdhury, PhD, Phang Boon Lim, MRCP PhD, Emmanuel Dupont, PhD, Steven A. Niederer, PhD, Chris D. Cantwell, PhD, Nicholas S. Peters, MRCP MD, Fu Siong Ng, MRCP PhD.

## Conflicts of interest

None declared.
